# Purpose, persistence, and progress: building the next generation of physician-scientists

**DOI:** 10.1172/JCI206886

**Published:** 2026-05-01

**Authors:** Cynthia Y. Tang

**Affiliations:** University of North Carolina MD-PhD Program, University of North Carolina at Chapel Hill, Chapel Hill, North Carolina, USA.

Each year, the Joint Meeting brings together the Association of American Physicians (AAP), the American Society for Clinical Investigation (ASCI), and the American Physician Scientists Association (APSA), three organizations with a long-standing commitment to scientific excellence, community building, and physician-scientist training. This year is particularly meaningful, as it is the APSA’s 20th Annual Meeting. It has been my honor to have served the APSA for the past four years, and now as President, it is my privilege to welcome everyone to the 2025 Joint Meeting.

## Discovering purpose

I would like to begin this address with a story. I was on a quiet flight to Vietnam. The cabin lights were dim. Everyone was wearing a mask. Every so often, a cough would break the silence. Sitting on my left, my mother was silently furious that we were traveling. On my right, my father was reading a newspaper with an image on the front cover of people in hazmat suits and headlines of a virus causing quarantines across Asia. This was 2003, at the end of the first SARS pandemic.

As a child, I was fascinated by how something so small could change the world. For the next few hours on the plane, I imagined myself inside one of those hazmat suits, fighting against the pandemic. Once we landed and the cabin doors opened, the humid air rushed in, palm trees in sight, and I promptly forgot all about this daydream.

Seventeen years later, in May 2020, history repeated itself. I had just taken US Medical Licensing Examination (USMLE) Step 1 during the COVID-19 lockdowns and was preparing to transition to PhD training in a flu lab. My new PhD mentor began a Zoom call by saying, “There’s a new virus. Any interest in working on it?” That was my chance to follow a childhood spark that I had, until then, forgotten. For the next four years, I studied the evolution, transmission, and clinical impacts of a novel pandemic virus, SARS-CoV-2.

In hindsight, the stories that we tell, on stage or in our publications, are always more polished than the actual journey, and in fact, the path is rarely linear. I reflect on my own winding journey to becoming a physician-scientist, how it parallels the growth of the APSA, and how our paths merged as we celebrate the APSA’s 20th Annual Meeting.

## Creating a road map

Like the APSA in its early years, I began without a clear road map. My parents immigrated to Idaho from Vietnam and have always been my biggest supporters in every dream I had growing up — from Olympic figure skater to astronaut to doctor. Yet for a first-generation college student, they could not provide practical guidance in navigating higher education, so I improvised.

I coached figure skating throughout high school and applied to colleges based on their proximity to ice rinks. I ended up choosing my college based on scholarship support and attended The College of Idaho, a beautiful liberal arts college that provided a wonderful space for me to explore my numerous interests in science, economics, philosophy, and music. I was surrounded by passionate mentors, and by graduation I had narrowed my interests to human health. At this point, the only doctors I knew were my pediatrician and dentist. I had never heard of a physician-scientist and could not imagine why anyone would spend almost a decade pursuing two doctorates.

This uncertainty is a common experience for many students.

**Figure 1:**
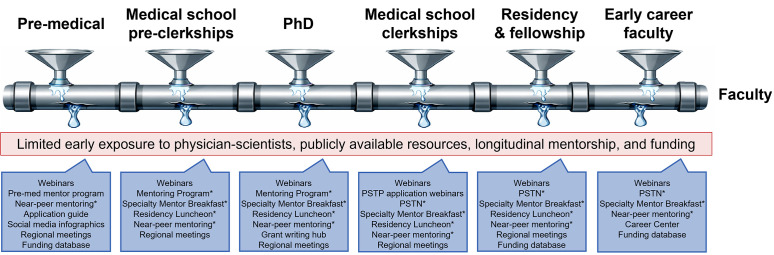
The leaky physician-scientist training pipeline and summary of interventions by the American Physician Scientists Association. Each pipeline segment represents a key transition point in physician-scientist training, corresponding to opportunities for recruitment and common points of attrition. The APSA offers training stage-specific resources across the continuum. The red bar denotes major causes of attrition, while the blue boxes highlight the APSA’s interventions at each stage. PSTN, Physician-Scientist Trainee Network; * indicates programming offered during the Joint Meeting.

In 2024, the APSA and the Burroughs Wellcome Fund convened the second Physician-Scientist Trainee Summit (1), bringing together trainees, program directors, and medical and scientific societies to identify barriers to entry and retention along the physician-scientist pipeline. This effort reflects a growing recognition that a diverse health care workforce is not only about opportunity but also about improving health outcomes for everyone (2–4). Participants collectively highlighted the same challenges that I once faced: limited early exposure to physician-scientists, a scarcity of accessible resources, and lack of longitudinal mentorship across each stage of training (Figure 1) (5). To address these systemic gaps, the APSA has increased early exposure through social media and webinars; expanded mentorship networks at the local, regional, and national levels across the training continuum; and developed tool kits for each key transition point along the pipeline.

## Finding the physician-scientist pathway

From my first encounter with a pandemic up through college, I had developed broad interests in health care, medicine, and discovery, but I didn’t know what to do with these interests until I met the right people. After college, I took a postbaccalaureate research fellowship at Washington University in St. Louis (WashU) studying cancer biology. I built a strong scientific foundation and realized that I preferred direct patient interactions and translational research.

I soon joined WashU’s Department of Anesthesiology as a clinical research coordinator, a job I hadn’t known existed. Over the next two years, I worked alongside clinician-scientists who taught me to ask meaningful questions, design studies, and translate data into better patient outcomes. This experience inspired me to pursue MD-PhD training. (Although when I very excitedly told one of my mentors that I planned to pursue an MD-PhD, he responded with great concern, “You don’t have to do this.” Fortunately, I did not follow that advice. He remains a close and valued mentor.)

For individuals like me, the APSA provides early awareness and access to the physician-scientist pipeline. The APSA offers live webinar initiatives including an annual Day in the Life of a Physician-Scientist, introduction to gap years and postbaccalaureate programs, and advice on applications and interviews for premedical trainees. We work to reach premedical offices at Historically Black Colleges and Universities (HBCUs), community colleges, and beyond. Additionally, as I learned throughout my own career, there are many types of research beyond traditional basic science and many pathways to become a physician-scientist. Accordingly, the APSA has broadened its scope to include colleagues in the social sciences, humanities, public health, and non–dual degree medical programs, recognizing that there are many ways to bridge science and medicine.

However, recruitment alone is not enough. Sustaining the workforce requires resources, mentorship, and community. There are multiple key transition points throughout physician-scientist training, and without proper resources and mentorship, these transition points also become exit points (Figure 1) (6, 7).

## Building community and resources

I began my MD-PhD training at the University of Missouri (Mizzou) in a small and relatively new program at the time. We received tremendous support and flexibility from our program leadership but had limited longitudinal infrastructure. It was so new that no one had graduated from the program yet. As I began to transition from preclinical to PhD training, especially as a nontraditional student pursuing bioinformatics research, I realized that I needed more resources and peer mentorship on how to navigate the training pathway. With encouragement from our program leaders, my peers and I founded a local APSA chapter in 2020. Through the APSA, I found friends across the nation who shared my values, mentors who shaped my goals, and opportunities I never even knew existed. With the combined support of my program leadership and peers from other institutions, I received my first grant, the first NIH F30 fellowship awarded at Mizzou.

Throughout this journey, I also met trainees at other institutions who faced similar challenges. Inspired by the support that I received, we formed a small team within the national APSA and launched our Grant Writing Hub in 2023, a national, crowdsourced repository of successful proposals, templates, and reviewer feedback — tools designed to level the playing field for trainees at newer or smaller programs. Led by passionate committee chairs, the APSA soon expanded our other databases containing postbaccalaureate research opportunities, social-science and humanities mentors, research-in-residency programs, and funding sources. In the past year, our Virtual Content Committee doubled our webinar offerings for current MD/DO-PhD students, expanding partnerships with organizations such as the Association of American Medical Colleges (AAMC). Over the past two years, the APSA has substantially expanded webinars for medical students, dual-degree students, residents, and fellows, including programming on research track residency programs, identifying mentors, grant writing, work-life balance, family planning, and board exams.

I have been fortunate to have incredible communities at Mizzou, the APSA, and now the University of North Carolina (UNC). For others who lack these networks, the APSA helps fill the gap, creating an enduring structure of shared knowledge and an expansive network of passionate individuals.

## Empowering mentorship

Mentorship is an essential component of physician-scientist training (7). The APSA’s longstanding virtual premedical mentorship program now matches between 250 and 350 mentor-mentee pairs each year. At the annual Joint Meeting, the Specialty Mentorship Breakfast connects trainees with faculty across disciplines. This year, we are piloting a new near-peer mentoring session, where trainees can seek mentorship from peers who have recently completed each step of training and learn to navigate the hidden curriculum. These mentoring topics range from MCAT preparation and surviving clerkships to family and financial planning and applying for faculty positions.

Additionally, in 2023, the APSA joined with the AAP and the ASCI to form the Physician-Scientist Trainee Network (PSTN), a longitudinal coaching program that pairs APSA members with AAP and ASCI mentors. This collaboration has become a highly anticipated Joint Meeting program for physician-scientist trainees nationwide, connecting medical students with longitudinal mentors, coaches, and sponsors as they move into residency and beyond.

## Celebrating 20 years of annual meetings

The 2025 Joint Meeting marks the APSA’s 20th Annual Meeting (8), and I reflect on how far we have come. In 2003, a small group of students, led by Freddy Nguyen, recognized a gap in trainee representation and filled it, creating the APSA and ultimately hosting the first national meeting in 2005. Today, the APSA serves more than 2,200 members at over 100 institutions worldwide and is continuing to grow.

This has been a monumental year for the APSA. A record number, more than 500 trainees from over 100 institutions globally, registered for the Joint Meeting. Over 70 AAP and ASCI members volunteered to mentor 270 students. At the residency luncheon, an opportunity for trainees to meet with program directors, a record of 34 residency programs participated. In addition to the generous awards provided by the AAP and ASCI, almost 60 travel awards were provided by the NIH, American Society for Investigative Pathology, Foundation for Anesthesia Education and Research, American Society of Nephrology, Society for Academic Emergency Medicine, American Association of Immunologists, Association of Medical School Pediatric Department Chairs, Burroughs Wellcome Fund, and the Texas Biomedical Research Institute to support trainee attendance. These numbers reflect a resilient, thriving physician-scientist community and a deep commitment to supporting it.

Our growth has been possible through steadfast partnerships. As the 2023–2024 APSA President, Alex Waldman, noted in his presidential address, “It takes a village” to create and sustain these initiatives (9). The AAP and ASCI welcomed the APSA’s community to their Joint Meeting in 2005 and fully integrated us in 2016 (8). We are also grateful to our generous partners, including the Lasker Foundation, the Burroughs Wellcome Fund, and the NIH, which have provided long-standing financial support to the APSA for our Joint Meeting sessions. Their generosity sustains the APSA’s travel awards, speaker series, and the vibrant community that gathers each year at the Joint Meeting. These partners have made this meeting an unmatched source of community, mentorship, and inspiration for physician-scientists-in-training.

I am reminded of my first Joint Meeting in 2021. Even virtually, for the first time I was surrounded by people who not only understood what it meant to be a physician-scientist but shared my passion for biomedical research, rather than being asked, “What is a physician-scientist?” or “Why would you do that?” I have attended every year since. Through the Joint Meeting, I have met inspiring leaders in academic medicine and industry, individuals who set strong examples of what a physician-scientist can accomplish. I have also made many close friends and mentors. Every year, I leave the conference with renewed motivation, inspiration, and affirmation that I am in the right place, and that I have chosen the best career in the world.

## Moving forward, together

As we continue into the next decade of the APSA, we face challenging times. The core of what we do, scientific inquiry, inclusive education, and equitable health care, is being questioned, restricted, and defunded (10). Yet our community persists. The record-breaking milestones outlined above highlight the commitment that physician-scientist leaders have for supporting the next generation and the resilience of the physician-scientist community. At this critical moment, we need everyone — trainees, mentors, institutions, industries, and organizations, not just as scientists and physicians, but as a collective community committed to scientific advancement, health equity, and forward progress. The APSA remains committed to creating a space where everyone is welcome and everyone can succeed.

Serving as the President of the American Physician Scientists Association has been the privilege of a lifetime. As I look ahead, I am reminded of the same lesson I learned as a child during the first SARS outbreak: during times of uncertainty, our passion, curiosity, and communities move us forward. With purpose, persistence, and progress, we will continue building the next generation of physician-scientists, together.

## Version 1 05/01/2026

Electronic publication
